# Supplementary material for: Elevated Likelihood of Infectious Complications Related to Oral Mucositis After Hematopoietic Stem Cell Transplantation: A Systematic Review and Meta-Analysis of Outcomes and Risk Factors
Source: Cancers (Basel). 2025 Aug 14;17(16):2657. doi: 10.3390/cancers17162657 (PMC12384372; doi:10.3390/cancers17162657)
Supplement: Supplementary file 1 [file cancers-17-02657-s001.zip › cancers-3760499-supplementary.pdf]

Supplementary Materials: Elevated Likelihood of Infectious Complications Related to Oral Mucositis After Hematopoietic Stem Cell Transplantation: A Systematic Review and Meta-Analysis of Outcomes and Risk Factors

Susan Eichhorn, Lauryn Rudin, Chidambaram Ramasamy, Ridham Varsani, Parikshit Padhi, Nour Nassour, Kapil Meleveedu, Joel B. Epstein, Benjamin Semegran, Roberto Pili and Poolakkad S. Satheeshkumar

Supplementary S1. Search Terms

("oral mucositis" [Title/Abstract] OR "mucositis" [Title/Abstract] OR "ulcerative mucositis" [Title/Abstract] OR "stomatitis" [Title/Abstract] OR "oromucositis" [Title/Abstract] OR "mucosal inflammation" [Title/Abstract] OR "mouth inflammation" [Title/Abstract] OR "mouth ulcer" [Title/Abstract] OR "oral ulcer" [Title/Abstract] OR "oral mucous membrane" [Title/Abstract] OR "mucous membrane" [Title/Abstract] OR "oral mucus membrane" [Title/Abstract] OR "mucus membrane" [Title/Abstract] OR "oral mucosa" [Title/Abstract]) AND ("stem cell transplantation" [Title/Abstract] OR "stem cell transplant" [Title/Abstract] OR "stem cell therapy" [Title/Abstract] OR "bone marrow transplantation" [Title/Abstract] OR "bone marrow transplant" [Title/Abstract] OR "bone marrow therapy" [Title/Abstract] OR "hematopoietic stem cell transplantation" [Title/Abstract] OR "hematopoietic stem cell transplant" [Title/Abstract] OR "hematopoietic stem cell therapy" [Title/Abstract] OR "HSCT" [Title/Abstract] OR "cord blood stem cell transplantation" [Title/Abstract] OR "cord blood stem cell transplant" [Title/Abstract] OR "cord blood stem cell therapy" [Title/Abstract] OR "mesenchymal stem cell transplantation" [Title/Abstract] OR "mesenchymal stem cell transplant" [Title/Abstract] OR "mesenchymal stem cell therapy" [Title/Abstract] OR "peripheral blood stem cell transplantation" [Title/Abstract] OR "peripheral blood stem cell transplant" [Title/Abstract] OR "peripheral blood stem cell therapy" [Title/Abstract] OR "cellular transplant" [Title/Abstract] OR "cellular transplantation" [Title/Abstract])

Supplementary S2. Case-Control Studies Bias Assessments

Study	*Selection*	*Comparison*	*Exposure*
Mikulska et al., 2010 [29]	★★★	★★	★★★
Lavallée et al., 2016 [28]	★★★	★★	★★★

Supplementary S3. Cohort Studies Bias Assessments

Study	*Selection*	*Comparison*	*Outcome*
Altes et al., 2007 [31]	★★★	★	★★★
Anaissie et al., 2004 [26]	★★★★	★★	★★★
Batlle et al., 2014 [32]	★★★	★★	★★★
Blijlevens et al., 2008 [33]	★★★	★★	★★★

Cho et al., 2019 [35]	★★★	★	★★★
Coleman et al., 2015 [18]	★★★	★★	★★★
Gebri et al., 2020 [36]	★★★	★★	★★★
Grazziutti et al., 2006 [38]	★★★	★★	★★★
Hong et al., 2020 [39]	★★★	★★	★★★
Kashiwazaki et al., 2012 [40]	★★★	★★	★★★
Kawamura et al., 2013 [41]	★★	★★	★★★
Laheij et al., 2012 [42]	★★★	★★	★★
Lee et al., 2018 [43]	★★★	★★	★★★
Lee et al., 2020 [44]	★★★	★★	★★
Legert et al., 2015 [45]	★★★★	★	★★
Nath et al., 2016 [47]	★★★	★	★★
Nguyen et al., 2015 [48]	★★	★★	★★★
Rocha et al., 2009 [49]	★★★	★★	★★★
Salvador, 2005 [50]	★★★	★	★★★
Santos et al., 2012 [30]	★★★	★★	★★
Shouval et al., 2019 [51]	★★★	★	★★
Sugita et al., 2012 [52]	★★★	★★	★★
Valeh et al., 2018 [53]	★★★	★★	★★★
Ursu et al., 2023 [55]	★★★	★	★★★
Saori Oku et al., 2022 [59]	★★★★	★	★★
Wong et al., 2022 [57]	★★★★	★	★★
Lachance et al., 2022 [60]	★★★	★	★★

Emine Merve Savas et al., 2024 [56]	★★★	★	★★
Khosroshahi et al., 2023 [54]	★★★	★	★★★
Deveci et al., 2022 [26]	★★★★	★★	★★

Supplementary S4. Randomized Controlled Trials Bias Assessments

Legert et al., 2015 [45]	🟢	🟢	🔴	🔴	🟢	🟢	🟢
Gori et al., 2007 [37]	🟢	🟢	🟡	🟡	🟢	🟡	🔴
Cho et al., 2017 [34]	🟢	🟢	🔴	🟡	🟢	🟡	🟢
	Random sequence generation	Allocation concealment	Blinding of participants and personnel	Blinding of outcome assessment	Incomplete outcome data	Selective reporting	Other Bias
